# AccuVIR: an ACCUrate VIRal genome assembly tool for third-generation sequencing data

**DOI:** 10.1093/bioinformatics/btac827

**Published:** 2022-12-26

**Authors:** Runzhou Yu, Dehan Cai, Yanni Sun

**Affiliations:** Department of Electrical Engineering, City University of Hong Kong, Kowloon, Hong Kong SAR 000000, China; Department of Electrical Engineering, City University of Hong Kong, Kowloon, Hong Kong SAR 000000, China; Department of Electrical Engineering, City University of Hong Kong, Kowloon, Hong Kong SAR 000000, China

## Abstract

**Motivation:**

RNA viruses tend to mutate constantly. While many of the variants are neutral, some can lead to higher transmissibility or virulence. Accurate assembly of complete viral genomes enables the identification of underlying variants, which are essential for studying virus evolution and elucidating the relationship between genotypes and virus properties. Recently, third-generation sequencing platforms such as Nanopore sequencers have been used for real-time virus sequencing for Ebola, Zika, coronavirus disease 2019, etc. However, their high per-base error rate prevents the accurate reconstruction of the viral genome.

**Results:**

In this work, we introduce a new tool, AccuVIR, for viral genome assembly and polishing using error-prone long reads. It can better distinguish sequencing errors from true variants based on the key observation that sequencing errors can disrupt the gene structures of viruses, which usually have a high density of coding regions. Our experimental results on both simulated and real third-generation sequencing data demonstrated its superior performance on generating more accurate viral genomes than generic assembly or polish tools.

**Availability and implementation:**

The source code and the documentation of AccuVIR are available at https://github.com/rainyrubyzhou/AccuVIR.

**Supplementary information:**

Supplementary data are available at *Bioinformatics* online.

## 1 Introduction

Many RNA viruses are able to generate new variants during their error-prone replication. Although many changes have little or no impact on the virus’ biological properties, some variants can gain undesirable functions such as increased transmissibility. There is evidence showing that viral strains in different subtypes, clades or subclades can have different biological functions or even incur different immune responses (Kim *et al.*, 2018), making accurate variant characterization an important step in studying viruses. For example, the omicron strain of severe acute respiratory syndrome coronavirus 2 (SARS-CoV-2) contains about 50 mutations in its genome compared to the delta strain, leading to enhanced transmission abilities (Tian *et al.*, 2022).

Obtaining accurate viral genomes from patients also provides important data for virus surveillance during epidemics (Hadfield *et al.*, 2018; Ladner *et al.*, 2019). For example, a popular strain-level analysis website, ‘Nextstrain’ (Hadfield *et al.*, 2018), organizes the virus genomes into subtypes, clades or subclades. These genome-based classifications help users track circulating strains during a pandemic such as coronavirus disease 2019 (COVID-19). The accuracy of the constructed genomes can directly affect the downstream analysis such as those at Nextstrain. Thus, there is a need for accurate construction of the viral genomes of various strains.

Whole-genome sequencing (WGS) is the major means to sequence viral strains. WGS can be conducted by next-generation sequencing (NGS) or third-generation sequencing (TGS) platforms. TGS’s main players, including Pacific BioSciences and Oxford Nanopore, can produce long reads covering whole genes or even whole virus genomes, making it highly amenable for reconstructing diverged or new strains. In addition, Nanopore provides real-time and portable genome sequencing platforms such as MinION devices, allowing on-site sequencing in rural or remote areas (Quick *et al.*, 2016). Another advantage of Nanopore for virus sequencing is that new technologies of direct RNA sequencing are emerging (Harel *et al.*, 2019; Seki *et al.*, 2019), which can remove reverse transcription or polymerase chain reaction bias. However, TGS data have a much higher per-base sequencing error rate than Illumina, the representative technology of NGS. Although PacBio Sequel II and the newest R10.4 Nanopore basecaller (https://nanoporetech.com/accuracy, date last accessed: March 21, 2022) can produce highly accurate (>99%) long reads (Wenger *et al.*, 2019), many TGS data, including some recently published datasets, do not reach the advertised accuracy (Amarasinghe *et al.*, 2020; Bull *et al.*, 2020; Harel *et al.*, 2019). In addition, these reads exhibit more insertion/deletion errors in homopolymers (Wenger *et al.*, 2019). For example, some recently published Nanopore datasets for WGS of SARS-CoV-2 have over 6% overall error rate and 8% error rate in low-complexity regions (Bull *et al.*, 2020). Thus, distinguishing true variants from sequencing errors remains challenging for viral genome assembly from long reads.

Although there are a number of error correction tools and assembly tools for TGS data (Kolmogorov *et al.*, 2019; Koren *et al.*, 2017; Li, 2016; Salmela *et al.*, 2017; Wang and Au, 2020), they are not optimized for viruses and cannot reach the desired accuracy for viral strains. Given the intractable errors in the assembly, there are some tools designed for polishing the assembled contigs. For example, Nanopolish (Loman *et al.*, 2015) takes fast5 files that contain raw signal of Nanopore sequencing data as input and generates polished consensus sequence based on a draft genome. It detects base variants using a hidden Markov Model (HMM). Medaka (https://github.com/nanoporetech/medaka) uses recurrent neural network (RNN) to predict correct bases at different positions. MarginPolish (Shafin *et al.*, 2020) uses a pair-HMM to generate a partial order alignment (POA) graph and iteratively polishes an assembly based on the graph. Utilizing weights of the final POA graph from MarginPolish, HELEN (Shafin *et al.*, 2020) further polishes the assembly by a multi-task RNN. Thus, MarginPolish and HELEN are often used together for polishing the assembly (Shafin *et al.*, 2020). Designed by the same team, a newer polisher based on RNN named PEPPER (Shafin *et al.*, 2021) is superior in both running time and performance. Although these tools can polish contigs from assembly tools, none of them is widely tested on viruses. A recently published polisher, Homopolish (Huang *et al.*, 2021), trained a support vector machine (SVM) model on homologous sequences to distinguish errors from true variants in an assembly. It was tested mainly on bacteria and a DNA virus but not on RNA viruses. When we applied Homopolish to RNA virus sequencing data, it could not retrieve enough homologous sequences and failed to polish the contigs.

In this article, we developed a tool called AccuVIR for the accurate assembly of viral genomes from TGS-sequenced WGS data. AccuVIR can be applied to either known or novel viruses to produce a viral genome with high accuracy. It achieves this goal by leveraging known error correction techniques such as the read alignment graph and the important observation that sequencing errors can disrupt gene finding outputs for viral genomes. Because RNA viral genomes are small and have high density of coding regions, the disruption of gene finding is more prominent in viruses than in other species. We tested our tool on both simulated and real TGS sequencing data and benchmarked it against popular TGS assembly and polish tools. The results show that our tool can produce high-quality viral genomes for different viruses and sequencing data of different sequencing coverage/average read length.

## 2 Materials and methods

**Fig. 1.**
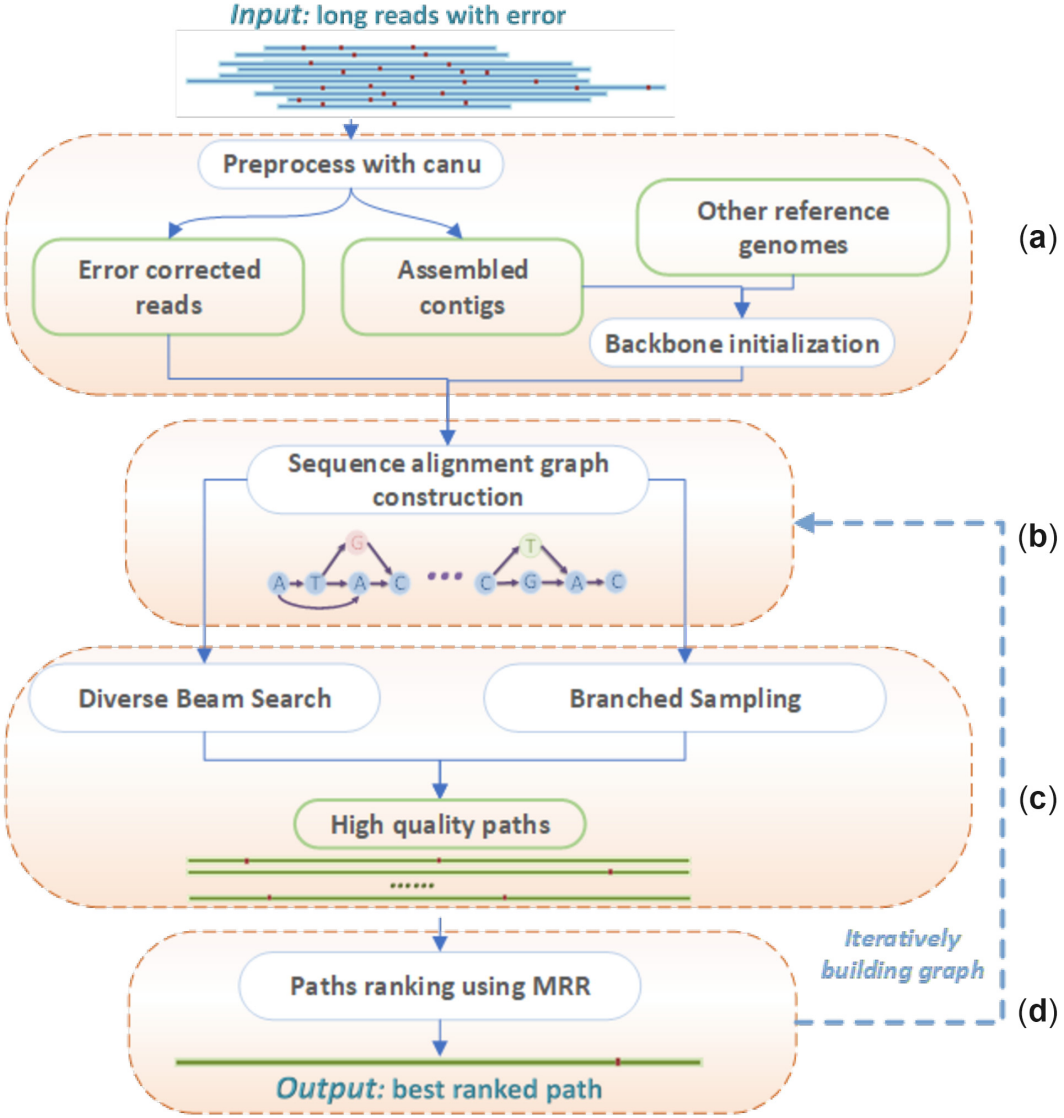
The pipeline of AccuVIR. (**a**) Preprocessing reads using Canu. AccuVIR allows users to use either the assembled contigs or a reference genome as the backbone for graph construction. (**b**) alignment graph construction. (**c**) Path sampling. (**d**) Path ranking

Sequencing errors can lead to fragmented gene prediction. Specifically, the insertion or deletion errors can disrupt correct reading frames and cause frameshifts, leading to lower gene density or shortened genes in computational gene finding. We thus integrate this key observation with other error-correction strategies in our method design. Figure 1 illustrates the key components of our method. First, we create a sequence alignment graph *G* using error-corrected reads by an error-correction tool (Fig. 1a). The default choice in our pipeline is Canu. Error correction tools can fail to correct all errors, especially those in low-complexity regions (such as homopolymer regions). Thus, the alignment graph summarizes the inconsistencies between different reads, consisting of both true variation and sequencing errors. In the constructed alignment graph, the correct genome is usually a path that is a concatenation of the error-free regions of different reads. Based on the edge weight information, we prefer paths with large path weights, indicating that many reads support those bases. In addition, this path should have high gene-finding scores, complete genes, etc. Because we choose the final path based on multiple criteria, we conduct path sampling in *G* first and then rank them accordingly. We sample two sets of paths. The first set contains paths with large path weights from a Diverse Beam Search (DBS). The second set contains sampled paths with slightly lower path weights but more variations (e.g. in homopolymer regions). These paths are candidate viral genomes (Fig. 1b). Then we apply gene finding and rank these genomes using multiple metrics, including the gene density, gene length, gene-finding score, etc. (Fig. 1c). Finally, we use mean reciprocal rank (MRR) to select and output the best one.

### 2.1 Iterative alignment graph construction

**Fig. 2.**
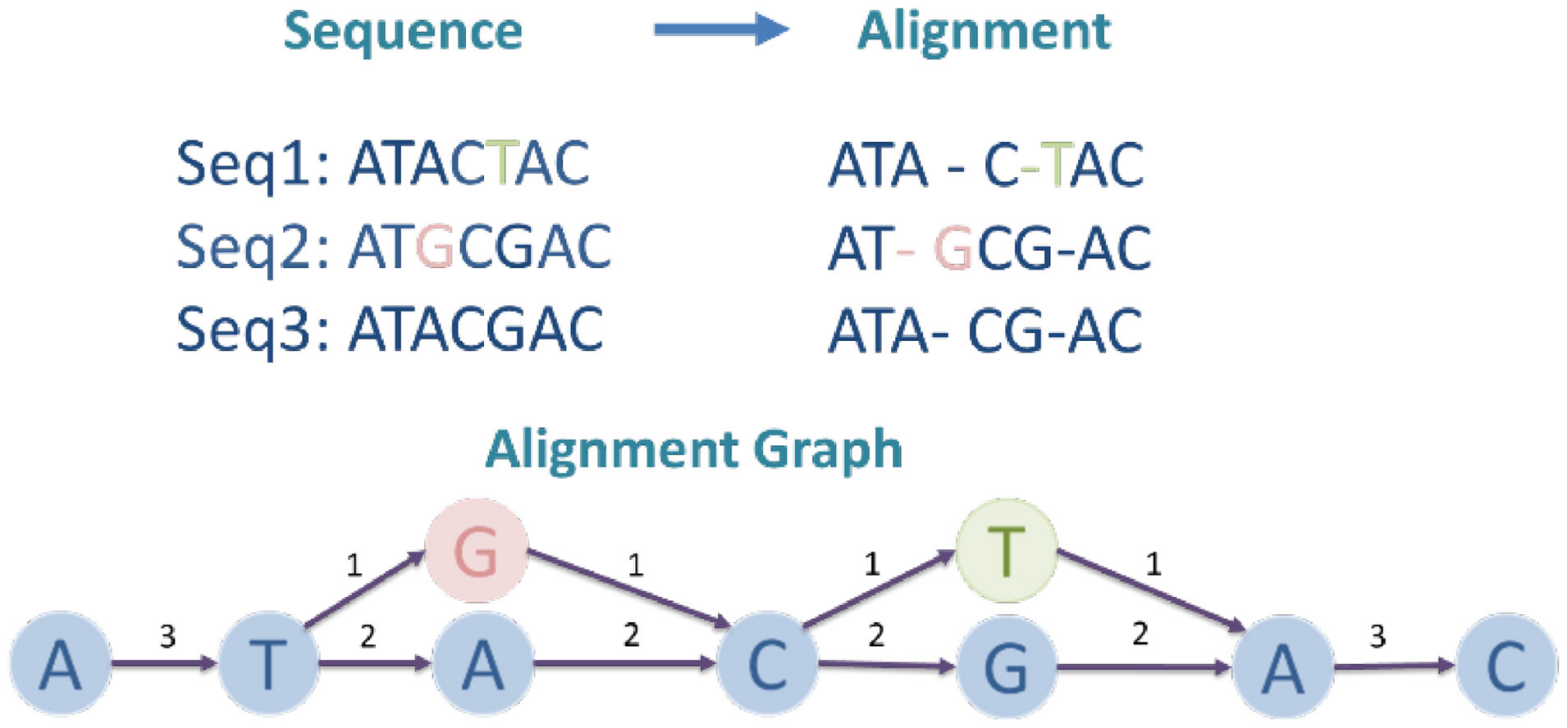
An alignment graph constructed from three sequences. Seq3 is used as the backbone to construct the sequence alignment graph

We follow the steps described in the hierarchical genome-assembly process (HGAP) (Chin *et al.*, 2013) for the alignment graph construction. A toy example of the graph is shown in Figure 2. We construct a directed acyclic graph (DAG) from input reads, denoted as G=(V,E). The nodes in *V* represent the bases in all error-corrected reads. If there is a read containing two consecutive bases denoted by nodes *v_i_* and *v_j_*, we add a weighted edge $\left(v_{i},v_{j}\right)$. The weight represents the number of reads that support the underlying edge.

To construct *G* efficiently, we choose a sequence as the backbone, which serves as a template for aligning other reads. Ideally, using a sequence that is closest to the viral haplotype of interest can lead to a graph with the minimum number of bifurcations caused by the differences between the true haplotype and the backbone sequence. However, without knowing the true haplotype, it is not trivial to pick the best template sequence. We provide two options. The first option is to use the assembled contig by an assembly program. The second option is to select one of the available reference genomes as the backbone if the target virus is known. This option is preferred if the output of the assembly programs is either too short or fragmented (i.e. containing many contigs). However, for RNA viruses with a high mutation rate, such as HIV-1 (Taylor *et al.*, 2008), the similarity between two different strains can be below 90%. To minimize the bifurcations in *G* incurred by the differences between the backbone and the real target genome, we introduce an iterative procedure to update *G* using a refined backbone, which is the output sequence of the preceding iteration (Fig. 1b–d). Users can decide the number of rounds to strike a balance between the total running time and the quality of newly generated sequences.

### 2.2 Path generation from sequence alignment graph

#### 2.2.1 DBS in alignment graph

In this step, we use a variation of Beam Search (BS) (Lowerre, 1976) to generate multiple candidate paths from the graph. BS explores the graph by always storing *B* most promising nodes in the search space and generating multiple best paths in the end. *B* is called the beam width. BS is equal to the best-first search when *B *=* *1.

**Fig. 3.**
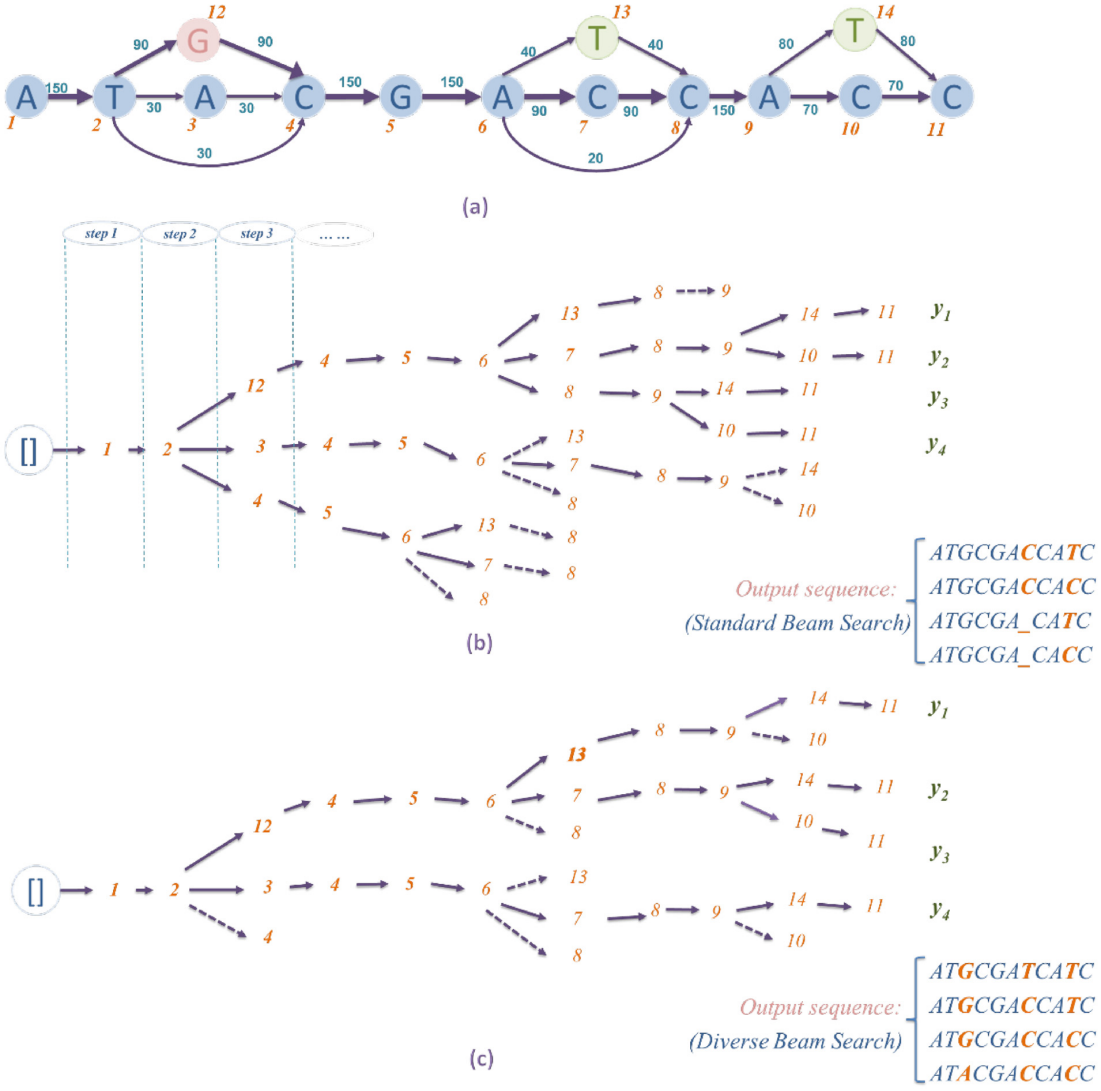
Comparison of Standard Beam Search (*B *=* *4) (**b**) and our Diverse Beam Search (*B *=* *2) (**c**) on an alignment graph (**a**). Solid arrows are the selected edges at each step. Dashed arrows are the discarded candidate edges at each step

To apply BS to an alignment graph, we start from a specified beginning node and initialize *B* empty paths as the output set ***Y***=[*y*_1_, *y*_2_,…,*y_B_*], where *y_i_* is the *i*th sequence. $y_{i}^{t}$ denotes the *t*th base in sequence *y_i_*. At each step (as annotated in Fig. 3b), we extend each output sequence by one base. For a node *v_x_*, we compute the extended path score $S(v_{x}|y_{b})$ for all path *y_b_* with *v_x_* as the succeeding node.

$$S(v_{x}|y_{b})=W(y_{b})+w(y_{b}^{t-1},v_{x})$$

$W(y_{b})$ is the path weight of *y_b_* and $w(y_{b}^{t-1},v_{x})$ is the edge weight from $y_{b}^{t-1}$ to *v_x_*.

One disadvantage of standard BS is that it lacks diversity in generated sequences. In our DAG, if one of the *B* paths has a dominating high score by step t, it is very possible that all its succeeding nodes will be selected for extending the path. As a result, these newly selected paths are identical in the first *t* − 1 bases. To address this problem, we adapted the methods developed by Vijayakumar (Vijayakumar *et al.*, 2016) and Li (Li and Jurafsky, 2016) to generate paths with better diversity. We use a new score that only considers the local weight for extending edges: $S^{\prime }(v_{x}|y_{b})=w(y_{b}^{t-1},v_{x})$. We use both *S* and $S^{\prime }$ to extend the paths. Also, the beam width of our search is expanded to 2*B*. To show the advantage of DBS, we compare the DBS of beam width *B* with standard BS of beam width 2*B*, which will both output 2*B* paths. In Figure 3b, standard BS (beam width = 4) stores diverse extending nodes at an early search stage from Step 3. However, from Step 7, it drops paths from other groups due to the large accumulated weight of a branch. On the contrary, in Figure 3c, because of the introduction of S′, DBS (beam width = 2) keeps nodes of other groups in Steps 7 and 9. In its output paths, DBS is able to cover all three variation sites correctly.

#### 2.2.2 Branched sampling in alignment graph

**Fig. 4.**
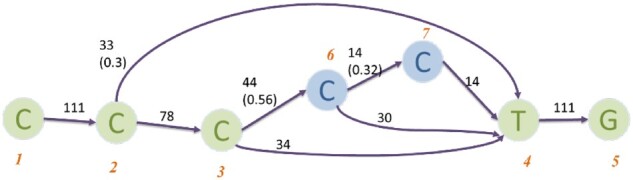
The alignment graph containing a homopolymer region. Node 1-5: backbone sequences. Node 6-7: insertions in read alignment

DBS in the previous stage can sample a set of paths with fewer errors. However, it is not optimized for handling errors in regions of low-complexity or low-sequencing coverage. For example, Nanopore reads have more insertion or deletion errors in homopolymer regions. Thus, some parts in the graph have more complex topology (especially homopolymer regions, see an example in Fig. 4).

**Fig. 5.**
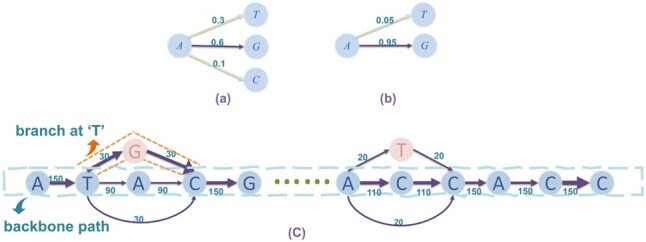
(**a**, **b**) With threshold *α *= 0.2, node ‘A’ in (a) is a candidate while node ‘A’ in (b) is not. (**c**) Branched sampling based on the backbone sequence

In order to embrace higher diversity in homopolymer regions, we design a branched sampling strategy. The basic idea of branched sampling is to replace original nodes of an input path using patches with high potential to represent the real variants. We define a patch as a short path with beginning and ending nodes on the given path but differs in the middle. In Figure 5c, string ‘TGC’ in orange square is a patch for the original ‘TAC’. We first select candidate starting nodes for branching by considering the second-largest weight of the outgoing edges. If the second-largest weight is bigger than a percentage (*τ*) of its total outgoing weights, it will be selected as a candidate starting node. Figure 5a and b shows an example. Second, we identify all homopolymer regions before branching starts. We first locate the end of a homopolymer (*v*_4_ for the example in Fig. 4) by finding nodes with a preceding node of the same base. Then, we trace back nodes in reverse topological order to locate the beginning of a homopolymer. Details of this algorithm are described in Supplementary Algorithm S1. Applying this algorithm to the example in Figure 4, we will get a node pair $\left(v_{1},\;v_{4}\right)$ as two ends of this homopolymer region.

Once we identify the candidate start nodes and the homopolymer regions, we will conduct branch sampling. For a start node *v_s_*, we take its largest-weight outgoing edge that is not on the original path (e.g. edge TG in the red box of Fig. 5c). This edge takes the original path to a new node v′ (e.g. the red node ‘G’ in Fig. 5c). To avoid including highly different paths with low weights, every additional extension after v′ uses the outgoing edge with the highest weight. We repeat the extension until a node on the original path is reached. If the start node *v_s_* is inside a homopolymer region, we apply a relaxed threshold (smaller *τ*) to generate more sampled patches. After we use all the start nodes, the patches are generated and will replace their corresponding subpaths in the original path. This process can be repeated again to generate a second alternative path with decreased *τ*. With the decrease of *τ*, the path diverges more from the original one (see Supplementary Fig. S2). Details of branched sampling can be found in Supplementary Algorithm S2. All the newly generated paths are passed to MRR for ranking.

### 2.3 Path ranking

Path sampling generates a set of sequences with relatively high quality. In the third step, we apply a gene-prediction tool (Hyatt *et al.*, 2010; Lukashin and Borodovsky, 1998) to examine the coding ability of a sequence, which provides us with additional information in distinguishing error-containing paths from the correct one.

**Fig. 6.**
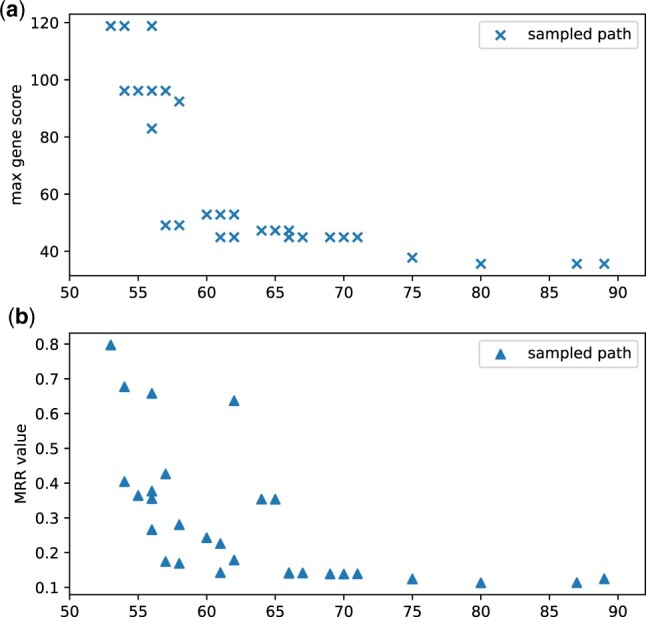
The change of sampled paths’ edit distances with the true genome versus (**a**) the gene-prediction scores from Genemark; (**b**) the MRR values; on simulated HIV-1 Nanopore data

Given a sequence, gene-prediction tools output all predicted coding regions with a score of each gene, usually in a general feature format (GFF) or general transfer format (GTF). Sequencing errors, particularly insertion or deletion errors, can disrupt gene finding and thus lead to low gene-finding scores. To examine this assumption, we sampled many paths from an HIV-1 sequencing dataset. Then, we calculated their edit distances with the true genome and the gene-prediction score, as shown in Figure 6a. The Spearman’s rank correlation coefficient (Kokoska and Zwillinger, 2000) of the edit distance and the gene-finding score is –0.88, indicating a strong negative relationship (–1 for the strongest) between edit distance and the gene-finding score. That means the edit distances between a sampled path and the correct viral genome generally decrease with the increase of the max gene score. Thus, utilizing max gene scores can provide a rough ranking of the sampled paths. However, only using the max score cannot make a finer distinction among individual paths because paths of different error rates can have the same max gene score. Thus, we need multiple gene-finding-related features to rank the paths with a higher resolution.

The first feature we use is the length of the path. If the target virus is known, we can rank the paths based on length differences between path lengths and approximate genome size. If it is a novel virus, we favor longer paths. The second feature we use is the number of coding regions found by gene-prediction tools. As indel errors lead to frameshifts in the translation process, errors will incur gaps in the predicted gene structure. For a given virus we are monitoring, the number of genes is usually stable in different variants. But sequences containing errors tend to have more genes with shorter lengths. Thus, we will rank the paths based on the gene structure. If the target virus is not known, we favor paths with fewer genes. The other two features are from the GFF output: max gene score and the total score of all genes.

After we rank the sampled paths using these features, we use the mean reciprocal rank method to select the best sequences as below.

(1) $$\text{MRR}=\frac{1}{N}\sum_{i=1}^{N}\frac{1}{{\text{Rank}}_{i}}.$$

Rank_*i*_ is the rank for a sequence in the *i*th feature. Based on the standard MRR, users can also assign weights to features to increase or decrease the importance of a feature. Calculated MRR values will provide an integrated score for the paths we have sampled. In Figure 6b, we present scatter plots of the MRR values and the edit distances of sampled paths on HIV-1 data. Compared with using a single feature such as max gene score to select the best path, MRR can better distinguish sequences with very few differences. Thus, we can select the path with the best MRR value as the most possible haplotype sequence. This helps us effectively screen the sampled paths and output the one with nearly the lowest error rate.

## 3 Experiments and results

We evaluated the performance of AccuVIR on both simulated and real TGS data. We conducted comprehensive tests on HIV, which has rapid and error-prone replication process and thus makes a suitable test case for viral genome assembly. We thus conducted experiments using both simulated Nanopore and PacBio data for HIV-1. We benchmarked AccuVIR against popular assembly and polish tools on these datasets. Then, we tested AccuVIR on real sequencing data in two groups of experiments. In the first group of experiments, we used real HIV-1 data with known variants. In the second group, we tested AccuVIR on SARS-CoV-2 datasets without known variants. We also tested AccuVIR on real Ebola sequencing data because Nanopore sequencing was applied to monitor the Ebola epidemic in several regions, and thus real data are more available. Due to the space limitation, the results of Ebola data are presented in Supplementary Section S4.1.

For all experiments with ground truth, we use QUAST (Gurevich *et al.*, 2013) to evaluate sequence quality. We present the errors in the coding regions and whole genomes, respectively. In addition, we also present edit distance to evaluate base-level quality. Details of the edit distance calculation can be found in Supplementary Section S1. For experiments with unknown ground truth, we focus on genome coverage and continuity, as well as the gene-finding scores in the evaluation.

### 3.1 Benchmark experiments on simulated data with different properties

#### 3.1.1 Experiments on simulated nanopore data

Because sequencing depth and average read length can significantly affect the performance of genome assembly, we simulated a series of Nanopore data from HIV-1 strain 89.6 with different attributes. Although there are several long read simulation tools such as NanoSim (Yang *et al.*, 2017), PBSIM (Ono *et al.*, 2013) and Badread (Wick, 2019), we choose Badread because it is more flexible in tuning the parameters such as sequencing depth and read length. The sequencing depths of the data are 50×, 100× and 200×, respectively. And the mean lengths are set as 2k, 4k and 6k, respectively.

We compare the performance of AccuVIR against different assembly and polish tools: Canu (Koren *et al.*, 2017), Flye (Kolmogorov *et al.*, 2019), viralFlye (Antipov *et al.*, 2022), metaFlye (Kolmogorov *et al.*, 2020), SPAdes (Bankevich *et al.*, 2012), rnaSPAdes (Bushmanova *et al.*, 2019), Strainline (Luo *et al.*, 2022), wtdgb2 (Ruan and Li, 2020), PEPPER (Shafin *et al.*, 2021), MarginPolish (Shafin *et al.*, 2020), HELEN (Shafin *et al.*, 2020), Medaka and PBDAG-Con (Chin *et al.*, 2013). The detailed versions or commit IDs we used for each tool are available in Supplementary Section S5.1. Among these tools, PEPPER, MarginPolish, HELEN and Medaka take in an assembled sequence from assemblers in their default pipelines. PBDAG-Con and AccuVIR also need a sequence as the backbone for constructing the read alignment graph. The input sequence can thus affect the performance of all tools. Considering that the assembled contigs may be fragmented, we conducted two groups of experiments. In the first group of experiments, we use a reference genome of the known virus as the input to all pipelines, which is preferred when the assembly programs fail to output long contigs. In the second group of experiments, we use the contigs from *de novo* assembly programs as input to all the tested tools.

Note that PBDAG-Con’s default graph construction uses the longest raw read. However, because the longest read is still too short in some datasets, we use our pipeline for backbone selection, which significantly improves the performance of PBDAG-Con. We did not include Nanopolish (Loman *et al.*, 2015) and homopolish (Huang *et al.*, 2021) in our experiment. The required signal data by Nanopolish is not available for most datasets. Homopolish failed to polish the input sequence, probably because it is not optimized for RNA viruses.

Table 1 presents the results where an HIV-1 reference genome is used as the input to tools requiring a draft sequence. Without making many assumptions, we adopted the most common strain HXB2 as the input reference. We measure the quality of the outputs by aligning the output sequence against the known strain. Specifically, we report the length of the largest alignment, the total aligned region and the unaligned region for alignment quality. Then, we present the number of mismatches, indels and total indel lengths in both coding regions and whole genomes for measuring the error patterns.

**Table 1.** Results on simulated HIV-1 datasets using HIV-1 HXB2’s genome as the input draft genome to all tools

Reads length	Depth	Tool	Largest align/total align	Unalign	Mis	Indels	Indel Len	Edit distance
2k	50×	PEPPER	9511/9511	265	4/22	26/105	30/174	277
		Margin	9694/9694	0	6/7	22/30	35/50	60
		Margin+HEL	8539/8539	889	5/13	19/54	32/89	518
		Medaka	9734/9734	0	12/14	12/16	22/44	54
		PBDAG-Con	9662/9662	0	1/1	39/41	46/48	52
		AccuVIR	**9700/9700**	**0**	**3/3**	**12/13**	**12/13**	**20**
	100×	PEPPER	9246/9246	0	9/14	40/97	52/153	185
		Margin	9711/9711	0	1/1	8/10	10/13	17
		Margin+HEL	9286/9286	295	0/14	15/52	20/79	224
		Medaka	9730/9730	0	6/9	14/17	25/34	46
		PBDAG-Con	9685/9685	0	0/0	17/18	23/25	28
		AccuVIR	**9707/9707**	**0**	**2/2**	**6/6**	**6/6**	**12**
	200×	PEPPER	9451/9451	132	29/15	16/58	28/106	154
		Margin	9709/9709	0	2/2	6/6	9/9	14
		Margin+HEL	9064/9064	503	0/2	5/11	12/18	199
		Medaka	9728/9728	0	8/10	11/13	20/26	38
		PBDAG-Con	9689/9689	0	0/0	17/18	19/21	24
		AccuVIR	**9703/9703**	**0**	**1/1**	**6/6**	**6/6**	**11**
4k	50×	PEPPER	9524/9524	0	7/11	59/100	84/167	187
		Margin	9699/9699	0	6/6	15/19	25/31	40
		Margin+HEL	9478/9478	133	2/20	39/88	51/123	186
		Medaka	9728/9728	0	8/12	15/19	37/48	62
		PBDAG-Con	9667/9667	0	0/0	32/35	39/43	46
		AccuVIR	**9700/9700**	**0**	**2/2**	**14/14**	**14/14**	**20**
	100×	PEPPER	9601/9601	0	7/18	28/66	44/131	149
		Margin	**9704/9704**	**0**	**1/1**	**8/8**	**12/12**	**16**
		Margin+HEL	9034/9034	594	6/61	25/30	40/53	211
		Medaka	9737/9737	0	8/14	10/13	21/29	46
		PBDAG-Con	9679/9679	0	0/0	23/24	30/31	34
		AccuVIR	9697/9697	0	0/0	12/13	13/14	20
	200×	PEPPER	9596/9596	0	57/9	33/70	51/118	130
		Margin	9700/9700	0	1/1	11/12	17/20	24
		Margin+HEL	9213/9213	380	1/2	15/18	21/25	136
		Medaka	9724/9724	0	6/10	10/14	17/26	38
		PBDAG-Con	9678/9678	0	0/0	23/24	31/32	35
		AccuVIR	**9708/9708**	**0**	**1/1**	**10/10**	**11/11**	**16**
6k	50×	PEPPER	9483/9483	0	9/17	48/132	88/245	261
		Margin	9702/9702	0	3/3	15/18	19/22	28
		Margin+HEL	9647/9647	0	18/23	51/67	59/84	119
		Medaka	6163/6163	3556	176/216	0/0	0/0	600
		PBDAG-Con	9660/9660	0	0/0	36/42	48/54	57
		AccuVIR	**9687/9687**	**0**	**0/0**	**17/18**	**21/22**	**26**
	100×	PEPPER	9650/9650	0	7/9	29/35	45/72	80
		Margin	9702/9702	0	1/1	17/17	24/24	28
		Margin+HEL	9696/9696	0	2/2	17/20	24/28	33
		Medaka	9722/9722	0	8/8	11/13	18/24	35
		PBDAG-Con	9683/9683	0	0/0	21/22	26/27	30
		AccuVIR	**9687/9687**	**0**	**2/2**	**8/8**	**8/8**	**26**
	200×	PEPPER	9671/9671	0	12/31	30/168	36/57	71
		Margin	**9711/9711**	**0**	**0/0**	**5/6**	**7/9**	**12**
		Margin+HEL	9709/9709	0	1/1	7/9	8/11	15
		Medaka	9724/9724	0	7/9	11/13	18/24	36
		PBDAG-Con	9674/9674	0	0/0	26/30	33/38	41
		AccuVIR	9704/9704	0	1/1	12/12	15/15	20

*Note*: PBDAG-Con uses the graph constructed by AccuVIR in these experiments for better results.

Largest align/total align, the ratio of the largest alignment’s length to the total alignment’s length. Unalign, the length of the unaligned region in the output sequence; Mis, number of mismatches; Indels, the number of gap opening; Indel Len, the total number of gaps; Edit Distance, the edit distanced between the output sequence and the ground truth sequence.The best result for each column is shown in bold.

**Fig. 7.**
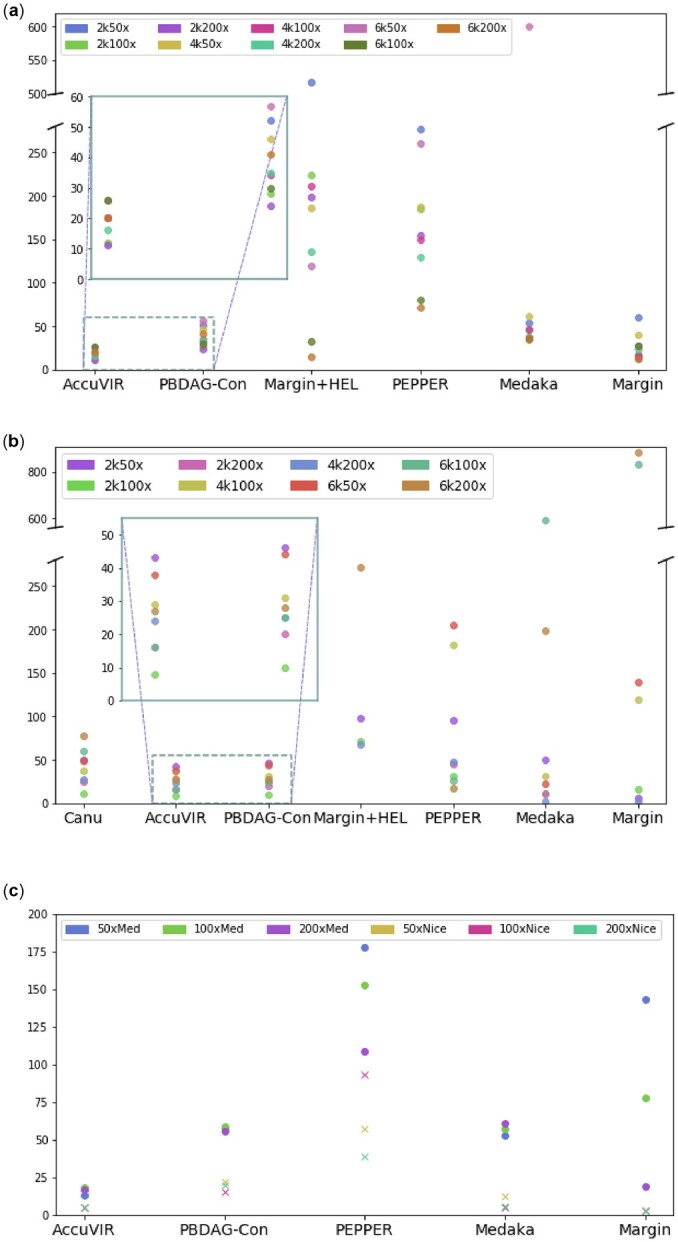
Quality of different polish tools’ outputs on the simulated HIV-1 datasets. Y-axis: edit distance between the output sequence and the known strain. X-axis: different tools. (**a**) Errors in different tools’ outputs using HXB2’s genome as the input sequence. (**b**) Errors in different tools’ outputs using Canu’s output assembly as the input sequence. (**c**) Errors in different tools’ outputs on simulated PacBio data of HIV-1. HXB2’s genome is used as the input sequence

With a complete reference genome as the input for generating the alignment graph, the outputs of all tools are relatively complete, reflected by their alignment lengths in Table 1. AccuVIR has the most robust performance across all these datasets with the edit distance ranging from 11 to 26. In addition, it constructs contigs with the smallest edits distance among all datasets except two. Its performance usually improves with the increase of the sequencing depth. In contrast, all learning-based polishers have large fluctuations (ranging from 12 to hundreds of errors) as visualized in the scatter plot of Figure 7a.

Due to space limitation, we show the detailed results of using assembly tools’ output as the input sequence in Supplementary Table S1. The error distributions of all tools can be found in Figure 7b. We first compared the performance of eight *de novo* assembly tools: Canu, Flye, viralFlye, metaFlye, SPAdes, rnaSPAdes, Strainline and wtdgb2. Flye and its two variants only work on two datasets with coverage greater than or equal to 200×. Both SPAdes and rnaSPAdes only have outputs for some of the datasets and always contain hundreds of errors. Strainline works for all datasets but generates sequences much longer than the genome on seven datasets. wtdbg2 works for most datasets except two with 2k read length, but its outputs are shorter than Canu. Canu is able to output an assembled contig for all datasets except the one with 50× coverage and 4k length. Overall, Canu’s assembly quality beats all other tools. Thus, we use Canu’s output as the input to other polish programs. As Supplementary Table S1 shows, AccuVIR has the most robust outputs across different datasets. It outputs the highest quality genome in five of them. In contrast, other tools have more variations (Fig. 7b). PBDAG-Con renders small mutation errors but still has more indels than AccuVIR. MarginPolish has the best performance in one dataset but much worse outputs on others.

#### 3.1.2 Experiments on simulated PacBio data

In this experiment, we simulated PacBio datasets of HIV-1 strain 89.6 with both mediocre (mean accuracy = 85%) and nice (mean accuracy = 95%) quality using Badread. Other parameters of the simulation are similar to Nanopore. The full set of details can be found in Supplementary Table S2. The draft sequence for all other tools is still the genome of HIV-1 strain HXB2. When the read quality is mediocre, AccuVIR is the only tool that generates less than 20 errors for datasets of all sequencing depths. When the mean read quality increases to 95% (with some reads’ accuracy >99%), all polishers’ results contain fewer errors. AccuVIR and Margin are the only two that generate sequences without any errors in aligned regions. The errors of different tools’ outputs are summarized in Figure 7c.

In Supplementary Section S4.3, we also show how MRR helps identify better paths from DBS’s output using the simulated HIV-1 PacBio data as an example.

### 3.2 Experiments on real HIV-1 virus sequencing data

It is difficult to find a real viral sequencing dataset with known variants. Thus, we used the PacBio sequencing data from a mock HIV-1 community (Giallonardo *et al.*, 2014). To create the datasets for viral genome reconstruction, we separate this dataset into read sets from five HIV strains. We first applied Canu for assembly. However, it outputs multiple short contigs. Thus, we choose HXB2 as the backbone for the alignment graph construction. We only show the case study of two sets of reads from strains 89.6 and JRCSF, which have lower similarity (93.88% and 94.61%, respectively) with the backbone sequence (HXB2) than the other two viral strains, thus making harder test cases. We tested PEPPER, MarginPolish, MarginPolish+HELEN, Medaka and PBDAG-Con on these two datasets. The results are summarized in Table 2. For the strain 89.6, AccuVIR generates a sequence of only 28 edit distance, ranking first. Among other tools, only Medaka and PBDAG-Con output sequences with edit distance below 100, but both have more mismatch or indel errors than AccuVIR. The case of JRCSF is trickier because the length of JRCSF is 179 bases shorter than the backbone (HXB2). Although all tools generate sequences with less than 51 errors in their aligned regions, the overall edit distances are all large because of the length difference between the target and the backbone sequence. Nevertheless, AccuVIR can identify a path closer to the target and thus has the smallest final edit distance of 118. Also, it has minimum errors in the aligned regions among all tools.

**Table 2.** Results on two real HIV-1 PacBio datasets

Strain	Backbone similarity (%)	Tool	Largest align/total align	Unalign	Mis	Indels	Indel Len	Edit distance
89.6	93.88	PEPPER	9590/9590	0	14/22	5/9	5/28	153
		Margin	9180/9180	333	13/15	7/7	8/8	247
		Margin+HEL	9121/9121	0	27/31	30/34	51/60	631
		Medaka	9684/9684	0	13/17	3/3	5/5	30
		PBDAG-Con	9701/9701	0	14/18	7/7	13/13	34
		AccuVIR	**9709/9709**	**0**	**12/16**	**6/6**	**8/8**	**28**
JRCSF	94.61	PEPPER	9191/9191	0	5/5	8/9	12/13	584
		Margin	9211/9211	187	3/3	8/9	13/14	399
		Margin+HEL	9172/9172	0	9/14	12/20	21/37	617
		Medaka	9515/9515	220	3/34	0/1	0/1	236
		PBDAG-Con	9525/9525	195	5/5	5/8	16/20	221
		AccuVIR	**9538/9538**	**111**	**3/3**	**0/1**	**0/1**	**118**

*Note*: The ground truth genomes for the two datasets are 89.6 and JRCSF, respectively. Strain HXB2 is used as the reference for AccuVIR and other polishers. PBDAG-Con uses the graph constructed by AccuVIR in these experiments for better results.The best result for each column is shown in bold.

### 3.3 Results on real SARS-CoV-2 sequencing data

Given the pandemic of COVID-19, it is of interest to test our method on its pathogen. In this experiment, we tested AccuVIR on five SARS-CoV-2 Nanopore sequencing datasets from nasopharyngeal swabs (SRX8723337–SRX8723341). Canu generated fragmented assemblies for these five datasets. For example, Canu’s best result is 10 short contigs with the maximum length of 3480 for the sample SRX8723338. Because these contigs are too short and fragmented, we used the first complete genome sequence of SARS-CoV-2, Wuhan-Hu-1 strain (29 903 bp), as the backbone to build our graph. We also compared the results of polishers that have good performance in the previous experiments: PEPPER, Medaka and PBDAG-Con. We present the edit distance between these tools’ outputs and the reference strain, as well as the gene-finding metrics (number of genes, total gene length and max gene length) in Table 3. On all five datasets, PEPPER and PBDAG-Con generated sequences that have edit distances between 65 and over 600 with the reference strain. Based on the continuous tracking of the mutations in SARS-CoV-2, these samples published in 2020 contain higher divergence than expected, indicating a high error rate. In contrast, Medaka and AccuVIR produced sequences with expected edit distances.

**Table 3.** Results on real SARS-CoV-2 Nanopore data

Dataset	Tool	Edit distance	len	No. of genes	sum len	max len
1	PEPPER	83	29 868	16	28 022	5972
	PBDAG-Con	494	29 519	13	28 079	6788
	Medaka	11	29 904	10	28 493	13 217
	AccuVIR	6	29 903	9	28 479	13 217
2	PEPPER	65	29 869	9	28 479	13 217
	PBDAG-Con	376	29 536	13	28 487	9416
	Medaka	13	29 902	9	28 479	13 217
	AccuVIR	2	29 904	10	28 538	11 879
3	PEPPER	514	29 682	29	24 250	2369
	PBDAG-Con	613	29 524	30	25 119	4361
	Medaka	69	29 934	17	27 838	10 661
	AccuVIR	3	29 903	11	28 333	13 217
4	PEPPER	367	29 729	25	26 708	3830
	PBDAG-Con	444	29 470	14	28 231	8675
	Medaka	19	29 909	11	28 504	13 235
	AccuVIR	8	29 904	9	28 470	13 217
5	PEPPER	404	29 803	28	25 553	2501
	PBDAG-Con	381	29 757	25	25 139	2786
	Medaka	31	29 917	12	28 302	7508
	AccuVIR	36	29 907	12	27 855	7157

*Note*: PBDAG-Con uses the graph constructed by AccuVIR in these experiments for better results.

Regarding the gene-prediction metrics, the total lengths of the predicted coding regions by different tools are close, usually having fewer than 100 bases difference. It is worth mentioning that the numbers of predicted genes are very different, with some tools generating more shorter genes. On datasets 1 and 4, the gene-finding results show that AccuVIR generated sequences covering 9 out of 12 coding regions in the genome. The missed coding regions are three short ones (E, ORF7b and ORF10). Medaka’s output also misses E, ORF7b and ORF10. In addition, it produces more fragmented gene products in ORF1ab in datasets 1, 3 and 4. An example of the alignment against ORF1ab is shown in Supplementary Figure S4. Specifically, we align Medaka’s and AccuVIR’s output sequences in dataset 3 against ORF1b of SARS-CoV-2. There are nine short local alignments in Medaka’s output. AccuVIR’s result generates three longer alignments with higher identity. Therefore, AccuVIR’s output is more consistent with current understandings of SARS-CoV-2.

## 4 Conclusion and discussion

Obtaining accurate variants (including mutations, insertions and deletions) in RNA viral strains is important for studying the biology of viruses. In this work, we presented a tool called AccuVIR that can assemble a high-quality viral genome from TGS-produced WGS data. It tackles the challenge of removing sequencing errors from TGS data by combining read alignment graph and the key observation that sequencing errors can disrupt gene-finding scores for RNA viruses, which have high density of coding regions.

Based on our experiments on simulated HIV-1 Nanopore data with different sequencing depths and read lengths in Supplementary Table S1, AccuVIR has the most robust outputs and consistently ranked top two among all tested tools/pipelines on different datasets. Some assembly polish tools that use machine learning methods to distinguish errors from true variants show more fluctuations on different datasets. For example, Medaka has indel errors ranging from only two to 96. PEPPER’s indel errors range from 16 to 400. MarginPolish+HELEN failed to output polished sequences on 3 out of 8 datasets and has indel errors ranging from 16 to 167. In contrast, AccuVIR has very stable output with indel errors between 10 and 48. Because many machine learning models have a large number of parameters and any domain shift in the training data and test data can cause unpredictable performance, this fluctuation is not surprising. Nevertheless, we did not see a clear trend that the learning-based models’ performance increases with the increase of the read length or sequencing coverage. Some of them have worse outputs for longer reads or high-coverage data. It is possible that these tools’ performance will improve if they are trained using RNA viruses with different sequencing conditions.

AccuVIR is designed for WGS data of viruses. It needs a relatively complete sequence as the backbone for constructing read alignment graph. Usually, for WGS data, the target virus is known, and thus users can choose to use the assembled contig or a known reference genome as the backbone. Using assembled contig has the advantage of obtaining a sequence that is close to the actual viral haplotype in the sample. However, assembly tools may not be able to assemble the reads into a relatively complete sequence, which is particularly problematic for low-coverage data and shorter reads. In this case, users can use a known reference genome as the backbone of AccuVIR. We tested the cases where the actual haplotypes have low similarity (e.g. 93.88%) with the backbone sequence. AccuVIR can still output a high-quality viral genome with a similarity of 99.82%.

By using AccuVIR to WGS data, it can help users identify the true variants with higher confidence. Because our tool is designed for RNA viruses, most of our experiments were conducted on Nanopore data. But it can be applied to both Nanopore and PacBio data.

## Supplementary Material

btac827_Supplementary_Data

## Data Availability

The tool of AccuVIR and the simulated data can be downloaded at https://github.com/rainyrubyzhou/AccuVIR. The source of real sequencing data are provided in corresponding sections.
