# Supplementary material for: AccuVIR: an ACCUrate VIRal genome assembly tool for third-generation sequencing data
Source: Bioinformatics. 2022 Dec 26;39(1):btac827. doi: 10.1093/bioinformatics/btac827 (PMC9825286; doi:10.1093/bioinformatics/btac827)
Supplement: btac827_Supplementary_Data [file btac827_supplementary_data.pdf]

AccuVIR: an ACCUrate VIRal genome assembly tool for third-generation sequencing data

Runzhou Yu, Dehan Cai and Yanni Sun

1 Edit distance calculation in the experiment results

Given a sequence S and a reference sequence R, we calculate the minimum number of edits (including deletion, insertion, and substitution) needed to transform S into R. This number is used as the edit distance in our evaluation metric.

However, in the benchmark experiment, many contigs Canu generated are two or three times longer than the target genome. To evaluate its results in a fair way, we want to evaluate only the region that contains the viral genome. We align the contigs to the backbone sequence that is used to build the graph and keep the longest aligned region for edit distance calculation.

2 Algorithms

Algorithm 1 Selection of homopolymer region

Input: Alignment Graph $G(V, E)$, V is topologically sorted
Output: Homopolymer regions $Homo\_loc$
Main Algorithm:

```
Homo_loc ← []
for v_i ∈ V do
    if frequency of base B in Prec(v_i)^a > 2 then
        homo_end ← v_i
        window ← V[i : i − 10]
        homo_begin ← succeeding node of first node that is
                     not base B in window
        append (homo_begin, homo_end) to Homo_loc
```

[a] Prec(v_i) is the set of preceding nodes for v_i.

Algorithm 2 Branching on a given path

Input: Alignment Graph $G(V, E)$;
Path $P[v_1, v_2, ..., v_n]$;
Candidate nodes set $CandiNodes[c_1, c_2, ..., c_m]$
Output: An alternative path based on P
Main Algorithm:

```
NewCandi ← []
for c_i ∈ CandiNodes and c_i ∈ P do
    patch^a ← [c_i]
    append argmax_{v_j ∈ Succ(c_i), v_j ∉ P} w_{i,j} to patch
    v_pend^b ← last node in patch
    while v_pend ∉ P do
        append argmax_{v_j ∈ Succ(v_pend)} w_{pend,j} to patch
        for v_k ∈ Succ(v_pend)^c do
            if v_k in homopolymer region then
                relax threshold for candidate selection
                if v_k is candidate then
                    append v_k to NewCandi
            end if
        end for
        Update v_pend
    end while
    v_pbeg^d ← first node in patch
    if v_pbeg and v_pend ∈ P then
        replace P[v_pbeg : v_pend] with patch
    end if
end for
Branching(G, P, NewCandi)
```

[a] A patch could be in the region that has been replaced by prior patches, will then ignore it.
[b] v_{pend} is the ending node in a patch.
[c] Succ(v_i) is the set of succeeding nodes for v_i.
[d] v_{pbeg} is the beginning node in a patch.

3 Supplementary Tables

Table S1: Results of different tools on simulated HIV-1 Nanopore datasets. Canu's output is used as input to all polish tools.
The best result for each column is shown in bold. "Largest align/Total align": the tool generating the largest alignment closest to the genome length is the best.
"Unalign": the tool with the shortest unaligned length is the best.
"Mismatch", "Indels", and "Indel Len": the tool with the fewest errors is the best.

Read length	Depth	Tools	Largest align/Total align	Unalign*	Mismatch*	Indels*	Indel Len
2k	50x	**Strainline**	8586/38694	0	37/42	200/231	255/290
		**SPAdes***	9066	-	159/198	320/405	-
		**rnaSPAdes***	-	-	-	-	-
		**metaFlye**	-	-	-	-	-
		**viralFlye**	-	-	-	-	-
		**Flye**	-	-	-	-	-
		**wtdbg2**	-	-	-	-	-
		**Canu**	9640/9640	0	0/0	46/50	59/63
		**PEPPER**	9265/9265	262	0/2	34/68	42/103
		**Margin**	**9704/9704**	**0**	**0/0**	**5/6**	**6/7**
		**Margin+HEL**	8965/8965	575	1/18	8/62	10/89
		**Medaka**	9640/9640	0	0/0	46/50	59/63
		**PBDAG-Con***	9651/9651	0	0/0	43/46	49/52
		**AccuVIR**	9696/9696	0	3/3	12/12	12/12
	100x	**Strainline**	6878/9459	0	9/9	43/47	51/55
		**SPAdes**	-	-	-	-	-
		**rnaSPAdes**	-	-	-	-	-
		**metaFlye**	-	-	-	-	-
		**viralFlye**	-	-	-	-	-
		**Flye**	-	-	-	-	-
		**wtdbg2**	-	-	-	-	-
		**Canu**	6469/11655	0	0/0	26/28	35/37
		**PEPPER**	6463/11650	0	2/4	39/59	50/76
		**Margin**	**6481/11684**	**0**	3/7	17/25	20/28
		**Margin+HEL**	6071/10974	546	15/15	66/69	92/98
		**Medaka**	6469/11655	0	0/0	26/28	35/37
		**PBDAG-Con**	6472/11665	0	0/0	22/22	27/27
		**AccuVIR**	6480/11673	0	**0/0**	**17/18**	**21/22**
	200x	**Strainline**	9711/19404	0	0/1	35/38	35/110
		**SPAdes**	9240	-	153/166	260/281	-
		**rnaSPAdes**	-	-	-	-	-
		**metaFlye**	8691/17105	838	1/1	29/29	29/29
		**viralFlye**	8691/17105	838	1/1	29/29	29/29
		**Flye**	8124/9132	27	0/0	15/15	15/15
		**wtdbg2**	112/586	6535	8/8	0/0	0/0
		**Canu**	9204/13368	0	0/0	43/48	54/61
		**PEPPER**	9181/12974	296	2/4	32/47	50/69
		**Margin**	9203/13386	15	34/75	24/129	26/180
		**Margin+HEL**	8118/10807	1962	23/23	62/62	83/83
		**Medaka**	9215/13391	12	13/13	24/27	29/32
		**PBDAG-Con**	9212/13393	**0**	0/0	31/32	35/36
		**AccuVIR**	**9231/13387**	31	**0/0**	**10/10**	**10/10**

Mismatch*, Indels*, Indel Len*: they are presented by the number of errors in the coding region / the total number of errors.
SPAdes* and rnaSPAdes*: the evaluation tool QUAST generate many short alignments that might be caused by lots of mismatch and indel errors for results of SPAdes and rnaSPAdes. To evaluate them in a fair way, we use BLAST to generate longer local alignments and present longest alignment, mismatches, and gapopens (equal to indel numbers).
PBDAG-Con* uses the graph constructed by AccuVIR in these experiments.

Read length	Depth	Tools	Largest align/Total align	Unalign*	Mismatch*	Indels*	Indel Len
4k	50x	**Strainline**	9680/20596	0	40/43	154/168	190/206
		**SPAdes**	9848	-	154/199	262/315	-
		**rnaSPAdes**	-	-	-	-	-
		**metaFlye**	-	-	-	-	-
		**viralFlye**	-	-	-	-	-
		**Flye**	-	-	-	-	-
		**wtdbg2**	9039/9039	1149	6/6	56/56	64/64
		**Canu**	-	-	-	-	-
		**PEPPER**	-	-	-	-	-
		**Margin**	-	-	-	-	-
		**Margin+HEL**	-	-	-	-	-
		**Medaka**	-	-	-	-	-
		**PBDAG-Con**	-	-	-	-	-
		**AccuVIR**	-	-	-	-	-
	100x	**Strainline**	9686/15383	135	0/0	32/40	34/42
		**SPAdes**	9816	-	172/177	341/355	-
		**rnaSPAdes**	-	-	-	-	-
		**metaFlye**	-	-	-	-	-
		**viralFlye**	-	-	-	-	-
		**Flye**	-	-	-	-	-
		**wtdbg2**	9155/9155	1081	0/0	38/38	45/45
		**Canu**	9674/27089	0	0/0	87/101	106/121
		**PEPPER**	9698/25250	1415	114/24	217/400	294/589
		**Margin**	83/150	20037	-/4	-/0	-/0
		**Margin+HEL**	-	-	-	-	-
		**Medaka**	9698/27146	5	3/5	46/55	52/61
		**PBDAG-Con**	9676/27112	0	**0/0**	77/83	90/96
		**AccuVIR**	**9702/27798**	**0**	3/3	**46/50**	**48/52**
	200x	**Strainline**	9726/43030	46	3/8	112/127	121/195
		**SPAdes**	9455	-	130/143	237/257	-
		**rnaSPAdes**	8539	-	196/230	332/339	-
		**metaFlye**	9052/9052	0	0/0	21/22	23/24
		**viralFlye**	9052/9052	0	0/0	21/22	23/24
		**Flye**	5723/5723	0	0/0	14/15	15/16
		**wtdbg2**	9160/9160	1211	1/1	30/30	40/40
		**Canu**	9680/9680	0	0/0	23/27	28/33
		**PEPPER**	9631/9631	0	1/1	23/42	30/54
		**Margin**	9715/9715	0	0/0	2/2	2/2
		**Margin+HEL**	9196/9196	416	1/2	14/16	20/22
		**Medaka**	**9712/9712**	**0**	**0/0**	**2/2**	**3/3**
		**PBDAG-Con**	9682/9682	0	0/0	22/25	27/31
		**AccuVIR**	**9714/9714**	**0**	1/1	10/10	11/11

Mismatch*, Indels*, Indel Len*: they are presented by the number of errors in the coding region / the total number of errors.
SPAdes* and rnaSPAdes*: the evaluation tool QUAST generate many short alignments that might be caused by lots of mismatch and indel errors for results of SPAdes and rnaSPAdes. To evaluate them in a fair way, we use BLAST to generate longer local alignments and present longest alignment, mismatches, and gapopens (equal to indel numbers).
PBDAG-Con* uses the graph constructed by AccuVIR in these experiments.

Read length	Depth	Tools	Largest align/Total align	Unalign*	Mismatch*	Indels*	Indel Len
6k	50x	**Strainline**	9678/17268	0	11/14	119/137	151/178
		**SPAdes**	9855	-	187/207	313/357	-
		**rnaSPAdes**	-	-	-	-	-
		**metaFlye**	-	-	-	-	-
		**viralFlye**	-	-	-	-	-
		**Flye**	-	-	-	-	-
		**wtdbg2**	9106/9106	1199	2/2	65/65	71/71
		**Canu**	9658/18680	15	0/0	79/93	94/110
		**PEPPER**	9691/18529	17	11/14	106/233	120/345
		**Margin**	94/94	13684	3/3	0/0	0/0
		**Margin+HEL**	-	-	-	-	-
		**Medaka**	9696/18754	15	2/2	**32/36**	**36/42**
		**PBDAG-Con**	9667/18698	15	**0/0**	69/80	81/92
		**AccuVIR**	**9699/18761**	**14**	4/4	40/48	40/48
	100x	**Strainline**	9690/9690	0	**0/0**	**21/22**	**22/23**
		**SPAdes**	-	-	-	-	-
		**rnaSPAdes**	9839	-	210/222	383/400	-
		**metaFlye**	-	-	-	-	-
		**viralFlye**	-	-	-	-	-
		**Flye**	-	-	-	-	-
		**wtdbg2**	9151/9151	1197	0/0	32/32	35/35
		**Canu**	9686/27701	6	0/1	98/106	133/161
		**PEPPER**	9686/18714	7658	0/2	57/70	66/81
		**Margin**	88/710	21676	6/16	0/0	0/0
		**Margin+HEL**	-	-	-	-	-
		**Medaka**	9706/22287	5572	346/686	73/38	87/57
		**PBDAG-Con**	9689/27149	6	0/0	62/64	75/77
		**AccuVIR**	**9707/27089**	**0**	3/4	34/36	35/37
	200x	**Strainline**	9748/19440	0	0/0	44/50	106/112
		**SPAdes**	8735	-	92/106	241/244	-
		**rnaSPAdes**	9832	-	155/157	295/330	-
		**metaFlye**	-	-	-	-	-
		**viralFlye**	-	-	-	-	-
		**Flye**	-	-	-	-	-
		**wtdbg2**	9177/9177	1210	0/0	21/21	22/22
		**Canu**	9681/18659	33	8/10	80/93	108/131
		**PEPPER**	9696/10312	7175	4/30	14/16	15/17
		**Margin**	9720/10303	8223	4/15	**7/7**	**7/7**
		**Margin+HEL**	9566/9566	6187	40/93	62/167	74/235
		**Medaka**	**9712/18742**	33	64/104	64/96	77/120
		**PBDAG-Con**	9682/18730	33	0/0	46/52	54/60
		**AccuVIR**	9705/18776	**32**	**0/0**	23/23	24/24

Mismatch*, Indels*, Indel Len*: they are presented by the number of errors in the coding region / the total number of errors.

SPAdes* and rnaSPAdes*: the evaluation tool QUAST generate many short alignments that might be caused by lots of mismatch and indel errors for results of SPAdes and rnaSPAdes. To evaluate them in a fair way, we use BLAST to generate longer local alignments and present longest alignment, mismatches, and gapopens (equal to indel numbers).

PBDAG-Con* uses the graph constructed by AccuVIR in these experiments.

Table S2: Results of different tools on simulated HIV-1 Pacbio datasets. Canu's output is used as input to all polish tools, PBDAG-Con, and AccuVIR.

Reads Quality	Depth	Tools	Largest align/ Total align	Unalign	Mismatch	Indels	Indel Len	Edit Distance
**Mediocre (87.5%)**	**50x**	**Canu**	**9703/9703**	**0**	**0/0**	**5/6**	**5/6**	**10**
		**PEPPER**	9618/9618	0	15/22	65/110	79/154	178
		**Margin**	9666/9666	0	8/23	60/94	72/114	143
		**Medaka**	9735/9735	0	14/15	15/20	22/35	53
		**PBDAG-Con***	9660/9660	0	1/1	17/21	38/54	58
		**AccuVIR**	9700/9700	0	0/0	2/3	2/7	13
	**100x**	**Canu**	9687/9687	0	0/0	2/2	2/2	26
		**PEPPER**	9595/9595	0	19/26	53/84	67/118	153
		**Margin**	9605/9605	0	1/2	26/48	30/57	78
		**Medaka**	9737/9737	0	7/7	19/23	36/47	57
		**PBDAG-Con**	9661/9661	0	3/3	18/21	41/53	59
		**AccuVIR**	**9696/9696**	**0**	**0/0**	**3/3**	**12/12**	**18**
	**200x**	**Canu**	9711/18775	22	0/0	4/6	12/14	9084
		**PEPPER**	9645/9645	0	5/18	23/62	30/90	109
		**Margin**	9714/9714	0	2/2	9/10	13/14	19
		**Medaka**	9734/9734	0	10/10	19/23	35/48	61
		**PBDAG-Con**	9662/9662	0	1/1	14/18	36/52	56
		**AccuVIR**	**9700/9700**	**0**	**0/0**	**2/2**	**11/11**	**17**
**Nice (95%)**	**50x**	**Canu**	9713/12801	0	0/0	1/1	1/1	3089
		**PEPPER**	9701/9701	0	2/3	20/31	31/51	57
		**Margin**	**9710/9710**	**0**	**0/0**	**0/0**	**0/0**	**3**
		**Medaka**	9719/9719	0	0/0	2/4	5/9	12
		**PBDAG-Con**	9693/9693	0	0/0	8/9	15/19	22
		**AccuVIR**	**9708/9708**	**0**	**0/0**	**0/0**	**0/0**	5
	**100x**	**Canu**	-	-	-	-	-	-
		**PEPPER**	9659/9659	0	9/11	20/49	31/77	93
		**Margin**	**9710/9710**	**0**	**0/0**	**0/0**	**0/0**	**3**
		**Medaka**	9712/9712	0	0/0	2/2	2/2	5
		**PBDAG-Con**	9698/9698	0	0/0	5/5	12/12	15
		**AccuVIR**	**9708/9708**	**0**	**0/0**	**0/0**	**0/0**	5
	**200x**	**Canu**	-	-	-	-	-	-
		**PEPPER**	9690/9690	0	3/8	16/23	19/28	39
		**Margin**	**9710/9710**	**0**	**0/0**	**0/0**	**0/0**	**3**
		**Medaka**	9713/9713	0	0/0	1/1	3/3	6
		**PBDAG-Con**	9693/9693	0	0/0	7/8	15/17	20
		**AccuVIR**	**9708/9708**	**0**	**0/0**	**0/0**	**0/0**	5

PBDAG-Con* uses the graph constructed by AccuVIR in these experiments.

4 Supplementary Figures

4.1 Results on real Ebola virus sequencing data

In this experiment, we test AccuVIR and PBDAG-Con on real Nanopore sequencing data of Ebola virus. The data was sequenced in Guinea and submitted to NCBI in 2015 (ERR1248093). Because the variant is unknown for this dataset, we only compare the length of the final output and genome coverage with respect to a reference Ebola genome from NCBI (NC_002549.1). Available assemblers cannot generate a complete contig on this dataset. Canu can only generate 12 segmented short contigs (See Fig. S1). Thus, we used a sequenced Ebola viral strain MK672825.1 as the backbone for AccuVIR. Table S3 presents the results of PBDAG-Con and AccuVIR, both of which have stable outputs according to our experiments on simulated data. As the comparison shows, AccuVIR outputs more complete contigs than PBDAG-Con does. Without a ground truth genome, we cannot provide meaningful evaluations for the polish tools and thus did not include them.

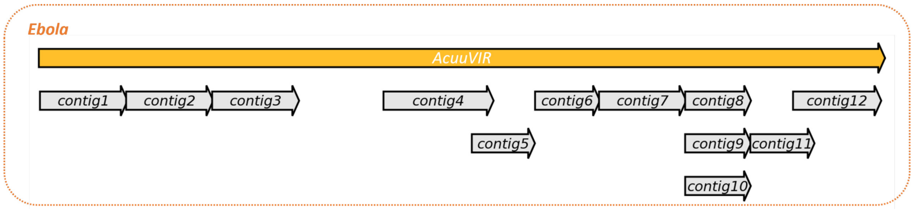

Figure S1: Assembly results of Canu and AccuVIR on real Ebola Nanopore data.

Table S3: Results of PBDAG-Con and AccuVIR on real Ebola Nanopore data

Dataset	Tool	Path Length	Genome Coverage(%)
**Ebola**	PBDAG-Con	18277	96.40
	**AccuVIR**	**18661**	**98.43**

PBDAG-Con uses the graph constructed by AccuVIR in the experiment for a better result.

4.2 Paths' quality with different τ

Our default value for the cutoff τ is determined empirically. Fig. S2 shows the change of the sampled paths' quality using different τ. Besides our default graph named *ec_ass graph*, which is constructed by aligning all error-corrected reads to Canu's assembled contig, we also tested τ using other graphs. In particular, we investigate how the method works if we use raw reads without error correction. *align n raw reads* means that the we align n raw reads in addition the error corrected reads to the backbone. We can see that when we add raw reads to the graph, the path quality will improve, most likely because the coverage (i.e. edge weight) becomes more accurate. However, the main caveat is that the graph size also increases significantly. And the edit distance is more volatile when we add raw reads. Thus, in our final implementation, we use error-corrected reads.

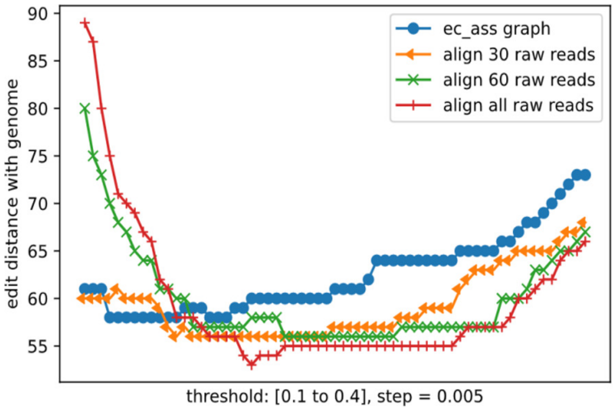

Figure S2: The change of sampled paths' edit distances with different τ on HIV-1 data. x-axis: threshold = $1 - \tau$;

4.3 Combination of Diverse Beam Search and MRR

Although Diverse Beam Search (DBS) generates a set of high-quality paths, a higher path score does not always lead to the fewest errors or the smallest edit distance with the target genome. As shown in Fig. S3 (a), there are many cases where paths with higher DBS scores have fewer errors despite the strong correlation. Combining DBS and the gene prediction score of these similar paths helps pinpoint the best path (Fig. S3 (b)).

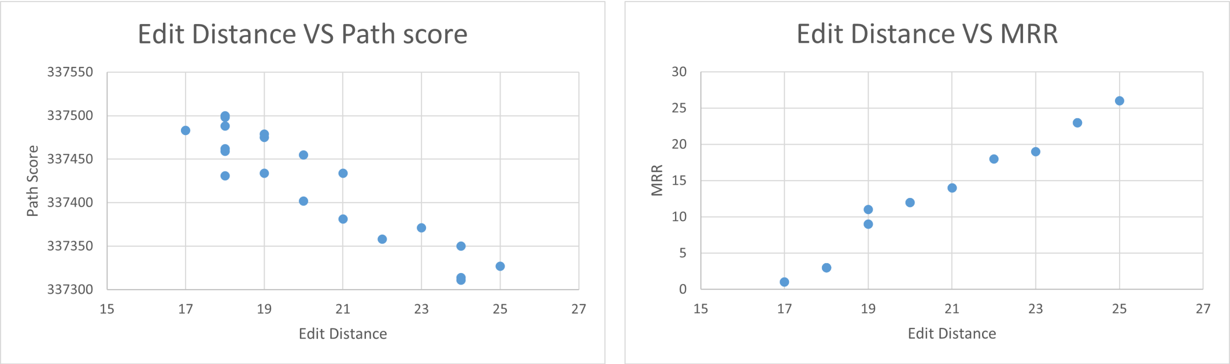

(a) Edit distance VS path score of generated paths in Diverse Beam Search. Correlation = -0.932.

(b) Edit distance VS MRR value of generated paths in Diverse Beam Search. Correlation = 0.989.

Figure S3: Effect of using gene finding score and MRR on path search in a simulated PacBio experiment. Each point corresponds to a sampled path.

4.4 BLASTX results on real SARS-CoV-2 Nanopore data

In Fig. S4, we align Medaka's and AccuVIR's output sequences from real dataset 3 against ORF1b of SARS-CoV-2 using BLASTX. There are nine short and fragmented local alignments in Medaka's output. In contrast, AccuVIR's result has three longer alignments on ORF1b, all with higher identities.

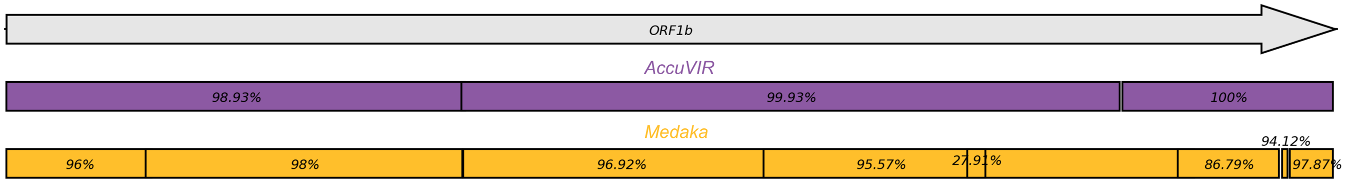

Figure S4: Alignment of Medaka's and AccuVIR's output sequences against ORF1b of SARS-CoV-2 (on real Nanopore dataset 3).

5 Tools and Experiment Commands

5.1 Versions for tools used in all experiments

- Strainline: -
- SPAdes: V3.14.1 rnaSPAdes V3.15.5
- Flye: V2.8.3 viralFlye: V0.2 metaFlye: V2.8.3
- wtdbg2: V2.5
- Canu: V2.1.1
- MarginPolish: V1.3.0
- HELEN: V0.0.23
- Medaka: V1.6.0
- PBDAG-Con: V0.2.3
- QUAST: V5.0.2

5.2 Commands

The following commands and parameters apply to HIV nanopore sequencing data. For tools requiring a genome size or the sequencing platform as an input, the parameters need to be modified accordingly for other viruses and other sequencing data.

Badread for simulating reads of different viruses:

Simulating Nanopore sequencing data:

```
badread simulate –reference virus_genome.fa –quantity 100x –error_model nanopore2020 > virus_100x_NP.fa
```

Simulating PacBio sequencing data:

```
badread simulate –reference virus_genome.fa –quantity 100x –error_model pacbio2016 > virus_100x_PB.fa
```

Badread for simulating reads of HIV-1 virus with different attributions:

```
badread simulate –reference HIV_ref.fa –quantity 50x –error_model nanopore2020 –length 2000,1000 > HIV_50x_2k.fa
badread simulate –reference HIV_ref.fa –quantity 50x –error_model nanopore2020 –length 2000,1000 > HIV_50x_4k.fa
badread simulate –reference HIV_ref.fa –quantity 50x –error_model nanopore2020 –length 2000,1000 > HIV_50x_6k.fa
badread simulate –reference HIV_ref.fa –quantity 100x –error_model nanopore2020 –length 4000,3000 > HIV_100x_2k.fa
badread simulate –reference HIV_ref.fa –quantity 100x –error_model nanopore2020 –length 4000,3000 > HIV_100x_4k.fa
badread simulate –reference HIV_ref.fa –quantity 100x –error_model nanopore2020 –length 4000,3000 > HIV_100x_6k.fa
badread simulate –reference HIV_ref.fa –quantity 200x –error_model nanopore2020 –length 6000,5000 > HIV_200x_2k.fa
badread simulate –reference HIV_ref.fa –quantity 200x –error_model nanopore2020 –length 6000,5000 > HIV_200x_4k.fa
badread simulate –reference HIV_ref.fa –quantity 200x –error_model nanopore2020 –length 6000,5000 > HIV_200x_6k.fa
```

Flye for running on HIV-1 data:

```
Flye –nano-raw reads.fa –out-dir 50x –genome-size 10k –threads 20
```

wtdbg2 for running on HIV-1 data:

```
wtdbg2 -x rs -g 10k -i reads.fa -t 16 -fo outdir/outname
wtpoa-cns -t 16 -i outdir/outname.ctg.lay.gz -fo outdir/outname.ec.cns.fa
```

Canu for running on HIV-1 Nanopore data:

```
Canu -d outdir -p outpref genomeSize=10k -nanopore reads.fa
```

PEPPER for running on HIV-1 data:

```
minimap2 -ax map-ont -t 32 draft_seq.fa reads.fa |samtools view -hb -F 0x904 > unsorted.bam
samtools sort -@32 -o reads.bam unsorted.bam
samtools index -@32 reads.bam
pepper polish –bam reads.bam –fasta draft_seq.fa –model_path pepper_r941_guppy305_microbial.pkl –output_file outfile –batch_size
128
```

MarginPolish for running on HIV-1 data:

```
minimap2 -ax map-ont -t 32 draft_seq.fa reads.fa | samtools view -hb -F 0x904 > unsorted.bam
samtools sort -@32 -o reads.bam unsorted.bam
samtools index -@32 reads.bam
python -m helen.marginpolish reads.bam draft_seq.fa models/MP_r941_guppy344_microbial.json -t 1 -o marginpolish_outdir -f
```

MarginPolish+HELEN for running on HIV-1 data:

```
helen polish –image_dir marginpolish_outdir –model_path models/HELEN_r941_guppy344_microbial.pkl –batch_size 256 –num_workers
4 –threads 8 –output_dir helen_outdir –output_prefix out_pref
```

Medaka for running on HIV-1 data:

```
medaka_consensus -i reads.fa -d draft_seq.fa -o out_pref
```

PBDAG-Con for running on HIV-1 data:

```
blasr reads.fa backbone.fa -bestn 1 -m 5 -out mapped.m5
pbdagcon mapped.m5 > output.fa
```

AccuVIR for running on HIV-1 data:

```
python AccuVIR_main.py -r reads.fa -b backbone.fa
```

(default beam width for Diverse Beam Search: 100;
different values for τ will be used, and all sampled paths will be passed to the next step.)

```
python AccuVIR_MRR.py -r paths.fa
```
